# Clinical Impact of Hospital-Acquired Anemia in Association with Acute Kidney Injury and Chronic Kidney Disease in Patients with Acute Myocardial Infarction

**DOI:** 10.1371/journal.pone.0075583

**Published:** 2013-09-24

**Authors:** Joon Seok Choi, Young A. Kim, Yong Un Kang, Chang Seong Kim, Eun Hui Bae, Seong Kwon Ma, Young-Keun Ahn, Myung Ho Jeong, Soo Wan Kim

**Affiliations:** 1 Division of Nephrology, Department of Internal Medicine, Chonnam National University Medical School, Gwangju, Korea; 2 Cardiovascular Research Institute of Chonnam National University, Gwangju, Korea; Scuola Superiore Sant'Anna, Italy

## Abstract

**Background:**

Hospital-acquired anemia (HAA) is common in patients with acute myocardial infarction (AMI) and is an independent indicator of long-term mortality in these patients. However, limited information exists regarding the development and prognostic impact of HAA associated with acute kidney injury (AKI) and chronic kidney disease (CKD) in AMI patients.

**Methods and Results:**

We retrospectively analyzed 2,289 patients with AMI, and excluded those with anemia at admission. The study population included 1,368 patients, of whom 800 (58.5%) developed HAA. Age, Hgb level at admission, Length of hospital stay, documented in-hospital bleeding and use of glycoprotein IIb/IIIa inhibitor, presence of CKD and occurrence of AKI were significantly associated with the development of HAA. HAA was significantly associated with higher 3-year mortality (4.8% and 11.4% for non-HAA and HAA patients, respectively; *P* < 0.001). After adjustment for multivariable confounders, the risk for long-term mortality was increased in HAA patients with AKI and/or CKD but not in HAA patients without AKI and/or CKD, compared to non-HAA patients (HAA patients without AKI and CKD, hazard ratio [HR]: 1.34, 95% confidence interval [CI]: 0.70–2.56; HAA patients with either AKI or CKD, HR: 2.80, 95% CI: 1.37–5.73; HAA patients with AKI and CKD, HR: 3.25, 95% CI: 1.28–8.24; compared with the non-HAA group).

**Conclusion:**

AKI and CKD were strongly associated with the development of HAA in AMI patients. HAA, when accompanied by AKI or CKD, is an independent risk predictor for long-term mortality in AMI patients.

## Introduction

Anemia, defined as reduced blood hemoglobin (Hgb) level, is common in patients with acute myocardial infarction (AMI), and is an independent indicator of in-hospital or long-term mortality in patients with AMI [1–4]. Considerable information exists regarding the effects of anemia in patients with AMI. However, few studies have focused on the effects of hospital-acquired anemia (HAA)—i.e. anemia developing during hospitalization in patients with normal Hgb levels at admission—on clinical outcomes after AMI [5,6]. Bleeding is one of the common non-cardiac complications in AMI patients. However, anemia can develop or worsen during hospitalization in the absence of overt bleeding [5,7]. Moreover, anemia is common among patients with chronic kidney disease (CKD), and is also frequently observed among patients who develop acute kidney injury (AKI) [8,9]. Failure of erythropoietin production to respond to decreased Hgb concentration appears to account for this observation [10]. A clear temporal relationship between reduced renal function and the decline in erythropoietin production and development of anemia has been documented [8–14].

Limited information exists on the role of renal disease in anemia in patients with MI, especially in HAA cases. The prognostic impact of HAA associated with renal disease has not been previously reported [2,5]. Because anemia and renal disease are independent risk factors affecting mortality in patients with AMI, understanding the role and prognostic implications of renal disease in HAA is important.

In the present study, we evaluated the risk factors for the development of HAA, especially in case of anemia in the setting of renal disease, and assessed the prognostic impact of HAA associated with renal disease in AMI patients.

## Subjects and Methods

### Ethics statement

The institutional review board of Chonnam National University Hospital, Gwangju, Republic of Korea approved this study. Given the retrospective design of the project, this institutional review board waived the need for consent. The study was performed in accordance with the Helsinki Declaration of 1975, as revised in 2000.

### Study population

A total of 2,289 patients admitted to the emergency department of Chonnam National University Hospital between January 2006 and October 2009 with a diagnosis of MI underwent initial retrospective review. We included both ST-segment elevated MI (STEMI) and non ST-segment elevated MI (NSTEMI) patients because pathophysiological process and cumulative in-hospital to long-term mortality did not differ between STEMI and NSTEMI patients [15–17]. Of these, 622 patients with anemia at the time of admission were excluded. We excluded an additional 285 patients who did not undergo percutaneous coronary intervention. Another 14 patients were excluded either because they did not undergo at least 2 Hgb measurements during hospitalization or because no follow-up data after discharge were available.

The final study population included 1,368 patients. Clinical characteristics as well as demographic, laboratory, and treatment data were obtained from the hospital’s computerized database. The diagnosis of MI was based on the triad of chest pain, electrocardiogram changes, and increased serum cardiac enzyme level [18]. Among MI patients, STEMI was defined by the presence of a new ST-segment elevation of at least 1 mm (0.1 mV) in continuous leads or a new left bundle-branch block on the index or electrocardiogram. Patients not classified as STEMI were considered to have NSTEMI based on the presence of positive biomarkers.

### Definitions

The Hgb value at admission was defined as the first Hgb value obtained during hospitalization. Anemia was defined by the World Health Organization (WHO) diagnostic criteria (Hgb < 13.0 g/dL in men, Hgb < 12.0 g/dL in women) [19]. HAA was defined as anemia developing during hospitalization in patients with normal Hgb levels at the time of admission. One trained study coordinator determined the presence of bleeding complications by reviewing the patients’ medical records.

Serial serum creatinine (SCr) levels during hospitalization, as obtained from the patients’ medical records, were reviewed by a trained study coordinator. Using the recently proposed standard KDIGO (Kidney Disease: Improving Global Outcomes) guideline, AKI was defined as either an absolute increase in the SCr level of ≥ 0.3 mg/dL within 48 hours, or an increase in the SCr level to ≥ 1.5 times the baseline that is known or presumed to have occurred within the previous 7 days [20]. Urine output was not considered as diagnostic criterion of AKI in this study. CKD was defined as an estimated glomerular filtration rate (eGFR) of < 60 mL/min/1.73m^2^ on admission [21]. The eGFR was calculated using the CKD-Epidemiology Collaboration (CKD-EPI) equation as follows: mL/min/1.73m^2^ = 141 × minimum (creatinine/κ, 1)^α^ × maximum (creatinine/κ, 1)^-1209^ × 0.993^age^ × 1.018 (if female) × 1.159 (if black); κ is 0.7 for women and 0.9 for men; α is -0.329 for women and -0.411 for men [22].

### Clinical end-points

The primary end-point of the study was 3-year mortality after MI. Survival after discharge from the hospital was determined from outpatient clinic records or from telephone interviews with patients or their relatives, physicians, and/or nursing home staff.

### Statistical analysis

Continuous variables were compared using Student’s *t* test and are presented as either mean (±SD) or median (with 25th and 75th percentiles). Categorical variables were compared using Pearson’s chi-square test or Fisher’s exact test and are expressed as numbers and percentages. The independent predictors of HAA were determined by logistic regression analysis. The variables analyzed included age, gender, body mass index (BMI), comorbidities (hypertension, diabetes mellitus [DM], ischemic heart disease [IHD], hyperlipidemia, and smoking status), Killip class, left ventricular ejection fraction (LVEF), Hgb level at admission, length of hospital stay, in-hospital bleeding, diagnosis (STEMI vs. NSTEMI), AKI, CKD, and medical treatments during hospitalization. To validate the impact of renal diseases such as AKI and CKD over HAA on mortality, we further categorized the study populations as follows: group I (n = 568), non-HAA patients; group II (n = 588), HAA patients without AKI and CKD; group III (n = 166), HAA patients with either AKI or CKD; group IV (n = 46), HAA patients with both AKI and CKD. The 3-year mortality was evaluated by the Kaplan-Meier method, and curves were compared using the log-rank test. The relationship between HAA and 3-year mortality was assessed by univariate and multivariate Cox proportional regression analyses. Multivariate analysis was performed for age, gender, BMI, comorbidities (hypertension, DM, IHD, hyperlipidemia, and smoking status), Killip class, LVEF, in-hospital bleeding, diagnosis (STEMI vs. NSTEMI), medical treatments during hospitalization, and categorized group according to presence of HAA, AKI and CKD. The hazard ratio (HR) and 95% confidence interval (CI) were reported for the Cox proportional model, and odds ratio (OR) and 95% CI were reported for the logistic regression model. All statistical tests were two-tailed, and P < 0.05 was considered significant. Statistical analysis was performed using the Statistical Package for Social Sciences software, version 18.0 (SPSS, IBM, Armonk, NY, USA).

## Results

### Baseline characteristics

The demographic data and baseline characteristics of the patients with and without HAA are shown in Table 1. The mean age in the total study population was 61.3 ± 12.4 years. Men constituted 77.0% of all patients. HAA developed during hospitalization in 800/1368 patients (58.5%). HAA was associated with older age, female gender, lower BMI, and a history of hypertension. At the time of admission, systolic and diastolic blood pressures were lower in HAA patients than in those without HAA. HAA patients presented with higher Killip class score and had a lower LVEF than non-HAA patients. Documented in-hospital bleeding was more common and mean length of in-hospital stay was longer in patients with HAA as compared to those without HAA. The proportion of STEMI did not significantly differ between patients with HAA and without HAA. HAA patients were also more likely to receive a glycoprotein-IIb/IIIa inhibitor and anticoagulant during hospitalization.

**Table 1:** Baseline characteristics of patients.

	Non-HAA (n=568)	HAA (n=800)	*P value*
Age, years	56.8 ± 11.7	64.5 ± 11.8	<0.001
Male (%)	487 (85.7%)	566 (70.8%)	<0.001
BMI, kg/m2	25.0 ± 3.1	24.2 ± 3.1	<0.001
**History**			
HTN	229 (40.3%)	366 (45.8%)	0.04
DM	129 (22.7%)	201 (25.1%)	0.30
IHD	69 (12.1%)	81 (10.1%)	0.24
Hyperlipidemia	31 (5.5%)	29 (3.6%)	0.10
Smoking	432 (76.1%)	498 (62.2%)	<0.001
**Initial presentation**			
SBP, mmHg	134 ± 27	130 ± 32	0.007
DBP, mmHg	83 ± 16	80 ± 19	0.005
HR, per minute	75.7 ± 17.4	74.3 ± 18.7	0.14
LVEF,%	57.5 ± 11.1	54.4 ± 12.3	<0.001
Killip class			<0.001
I	494 (87.0%)	621 (77.6%)	
II	47 (8.3%)	77 (9.6%)	
III	15 (2.6%)	63 (7.9%)	
IV	12 (2.1%)	39 (4.9%)	
**In-hospital course**			
Length of stay, days	6.3 ± 2.8	9.6 ± 10.9	<0.001
In-hospital bleeding	28 (4.9%)	110 (13.8%)	<0.001
**Diagnosis**			0.051
STEMI	353 (62.1%)	538 (67.2%)	
NSTEMI	215 (37.9%)	262 (32.8%)	
**In-hospital medication**			
Aspirin	566 (99.8%)	798 (99.8%)	0.63
Clopidogrel	567 (99.8%)	797 (99.6%)	0.20
Glycoprotein IIb/IIIa inhibitor	194 (34.2%)	339 (42.4%)	0.002
Anti-coagulant	567 (99.8%)	794 (99.2%)	0.04
Beta-blocker	503 (88.7%)	703 (87.9%)	0.64
Calcium channel blocker	30 (5.3%)	56 (7.0%)	0.20
ACEi or ARB	525 (92.4%)	720 (90.0%)	0.12
Statin	434 (76.5%)	592 (74.0%)	0.28

Abbreviations: HAA, hospital acquired anemia; BMI, body mass index; HTN, hypertension; DM, diabetes mellitus; IHD, ischemic heart disease; SBP, systolic blood pressure; DBP, diastolic blood pressure; HR, heart rate; LVEF, left ventricular ejection fraction; STEMI, ST-segment elevated MI; NSTEMI, non ST-segment elevated MI; PCI, percutaneous coronary intervention; ACEi, angiotensin converting enzyme inhibitor; ARB, angiotensin receptor blocker

### Changes in Hgb level and prevalence of AKI/CKD

Table 2 shows Hgb level changes during hospitalization and the prevalence of AKI and CKD. The Hgb level was more frequently measured during hospitalization in patients with HAA. The Hgb level at admission was 14.2 ± 3.3 g/dL in the HAA patient group and 15.3 ± 1.2 g/dL in the non-HAA patient group (*P* < 0.001). The lowest Hgb level during hospitalization was 11.3 ± 1.2 g/dL in the HAA patient group and 14.2 ± 4.2 g/dL in the non-HAA patient group (*P* < 0.001). The difference in Hgb level between the HAA patient group and non-HAA patient group persisted at the time of discharge (11.8 ± 1.2 vs. 14.2 ± 1.1, respectively; *P* < 0.001). The HAA patient group had a lower eGFR on admission, and a higher proportion of CKD. Moreover AKI occurred more frequently in HAA patients than in non-HAA patients.

**Table 2:** Hemoglobin changes and prevalence of AKI/CKD.

	Non-HAA (n=568)	HAA (n=800)	*P value*
**Hemoglobin level (g/dL**)			
Admission	15.3 ± 1.2	14.2 ± 3.3	<0.001
Nadir	14.2 ± 4.2	11.3 ± 1.2	<0.001
Discharge	14.2 ± 1.1	11.8 ± 1.2	<0.001
Number of Hgb measurements	3.1 ± 1.6	5.7 ± 6.2	<0.001
**Prevalence of AKI and CKD**			
eGFR on admission	88 ± 19	80 ± 21	<0.001
CKD	36 (6.3%)	143 (17.9%)	<0.001
AKI	26 (4.6%)	118 (14.8%)	<0.001
AKI stage			<0.001
0	542 (95.4%)	682 (85.2%)	
1	22 (3.9%)	80 (10.0%)	
2	2 (0.4%)	24 (3.0%)	
3	2 (0.4%)	14 (1.8%)	

Abbreviations: Hgb, hemoglobin; HAA, hospital acquired anemia; eGFR, estimated glomerular filtration rate; CKD, chronic kidney disease; AKI, acute kidney injury

### Independent predictors of HAA

Multivariate logistic regression analysis was used to identify significant predictive factors for the development of HAA (Table 3). Multivariate analysis showed that age (OR: 1.04, 95% CI: 1.02–1.05), Hgb level at admission (OR: 1.71, 95% CI: 1.52–1.94), length of hospital stay (OR: 1.23, 95% CI: 1.17–1.29), documented in-hospital bleeding (OR: 1.98, 95% CI: 1.17–3.33), and use of glycoprotein IIb/IIIa inhibitor (OR: 1.63, 95% CI: 1.22–2.17), were significantly associated with the development of HAA, following multivariate adjustment. In addition, the presence of CKD (OR: 1.92, 95% CI: 1.14–3.23) and occurrence of AKI (OR: 1.94, 95% CI: 1.02–3.67) were also associated with the development of HAA.

**Table 3:** Mutivariable logistic analysis of associated factor for HAA development.

	Odds ratio	95% confidence interval	*P* value
Age per year increase	1.04	1.02–1.05	<0.001
Admission Hgb level per mg/dl decrease	1.71	1.52–1.94	<0.001
Length of hospital stay per day increase	1.23	1.17–1.29	<0.001
In-hospital bleeding	1.98	1.17–3.33	0.01
glycoprotein IIb/IIIa inhibitor	1.63	1.22–2.17	0.001
AKI	1.94	1.02–3.67	0.04
CKD	1.92	1.14–3.23	0.01

The confounders analyzed included age, gender, BMI, comorbidities (hypertension, DM, IHD, hyperlipidemia, and smoking status), Killip class, LVEF, Hgb level at admission, length of hospital stay, in-hospital bleeding, diagnosis (STEMI vs. NSTEMI), AKI, CKD and medical treatments during hospitalization.

### Clinical outcomes of HAA in association with AKI and/or CKD

**Figure 1:**
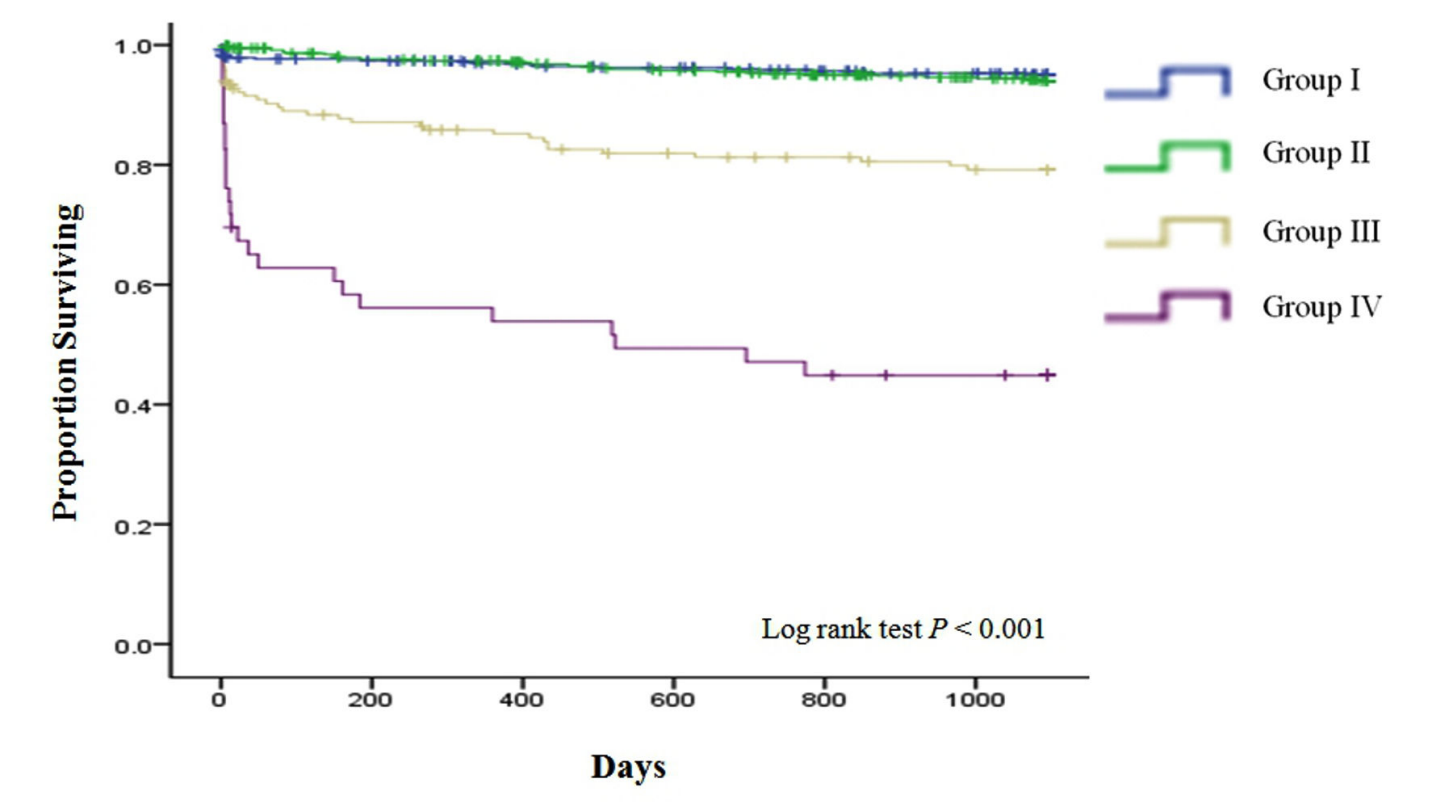
The 3-year cumulative survival rate of HAA patients with or without AKI and CKD. group I= non-HAA patients; group II= HAA patients without AKI and CKD; group III= HAA patients either AKI or CKD; group IV= HAA patients with both AKI and CKD.

The patients were followed-up for a mean period of 3.93 ± 1.81 years. For Kaplan-Meier and Cox-proportional regression analysis, the duration of follow-up was right-censored at 3 years. A total of 118 (8.6%) patients died during the follow-up period. HAA was significantly associated with higher 3-year mortality (3-year mortality rates: 4.8% and 11.4% for non-HAA and HAA patients, respectively; *P* < 0.001). To validate the comparative impact of renal diseases such as AKI and CKD over HAA on mortality, we further categorized HAA patients according to the presence of AKI and/or CKD. Kaplan-Meier curves showed significant differences in 3-year mortality among the 4 groups (Figure 1). Compared with groups I and II, groups III and IV were associated with higher 3-year mortality (log rank *P* < 0.001). There was no significant difference in 3-year mortality between groups I and II. Multivariate Cox proportional regression analysis was performed to evaluate the risk of death among the groups (Table 4). Using group I as a reference, no unadjusted or multivariable-adjusted association between group II and mortality was identified. In contrast, groups III and IV were strongly associated with mortality, even after multivariable adjustment (OR: 1.34, 95% CI: 0.70–2.56 for group II; OR: 2.80, 95% CI: 1.37–5.73 for group III; and OR: 3.25, 95% CI: 1.28–8.24 for group IV; compared with the non-HAA group).

**Table 4:** Multivariable association between HAA and 3-year mortality related with presence of AKI and/or CKD.

	Hazards ratio	95% confidence interval	P
Group I (n=568)	Reference	Reference	
Group II (n=588)	1.34	0.70–2.56	0.37
Group III (n=166)	2.80	1.37–5.73	0.005
Group IV (n=46)	3.25	1.28–8.24	0.01

The confounders analyzed included age, gender, BMI, comorbidities (hypertension, DM, IHD, hyperlipidemia, and smoking status), Killip class, LVEF, in-hospital bleeding, diagnosis (STEMI vs. NSTEMI), and medical treatments during hospitalization.

group I = non-HAA patients; group II = HAA patients without AKI and CKD; group III = HAA patients either AKI or CKD; group IV = HAA patients with both AKI and CKD.

## Discussion

Our retrospective study of AMI patients who had a normal Hgb level at admission demonstrates that renal diseases such as AKI and CKD are associated with the development of anemia. This study further indicates increased mortality among patients who developed anemia during hospitalization. Subgroup analysis revealed that renal disease, such as AKD and/or CKD, in HAA patients was strongly associated with poor outcome, and remained an independent risk predictor of long-term mortality after adjustment for multivariable confounders. There was also a graded increase in the risk of death depending on whether or not AKI and CKD were present alone or together in this setting. HAA patients without renal disease had no increase in long-term mortality compared to non-HAA patients. These observations are vital because they highlight the important role of AKI and CKD in the development of HAA, and also suggest that AKI and CKD are related to increased risk of death for AMI patients who develop anemia during hospitalization. The mortality risk for HAA in association with AKI and CKD has not been previously reported, and the present study findings extend previous insights on the role and impact of HAA on the clinical outcomes in AMI patients.

In the present study, we observed that more than half the patients with normal Hgb levels at admission developed anemia during hospitalization. Previous studies reported an incidence of HAA of 45–57% [5,6,23]. Although we noted a slightly higher incidence in our study, as compared to previous reports, different definitions of anemia and HAA were used. The etiology of anemia in AMI patients is likely to be multifactorial. Our study population excluded patients with anemia at admission to eliminate the factors associated with chronic anemia. Notably, we found a strong association between AKI and CKD, and the development HAA, as shown by the analysis of multivariable confounders. Anemia among patients with chronic kidney disease primarily occurs due to abnormal erythropoietin activity. Recent evidence also indicates that uremia-induced inhibitors of erythropoiesis, nutritional deficiencies, shortened erythrocyte survival, and disordered iron homeostasis also contribute to the development of anemia in these populations [24]. Anemia is also very common in patients with AKI, and Hgb levels decrease rapidly in the first few days of AKI, followed by profound and protracted anemia [9,12]. This may be explained by several mechanisms. Many patients with AKI already had signs of severe inflammation, resulting from the direct effect of inflammatory cytokines. Anemia is also, at least in part, a marker of hemodilution, and may develop due to consequence of volume expansion in hemodynamically unstable patients [25]. In addition, anemia in patients with AKI is a consequence of increased red blood cell destruction and loss, or decreased production of red blood cells [26]. Our results are consistent with those of previous studies, and are related to a specific population, i.e., AMI patients with normal Hgb levels at admission [8,9,13].

The present study shows that HAA in the absence of AKI and CKD was not an independent predictor of long-term mortality in patients with AMI. In contrast, HAA patients with AKI and/or CKD had a significantly poorer outcome, and this combination of risk factors was an independent predictor of long-term mortality after adjustment for multivariable confounders. Our findings add an important new perspective to previous reports concerning the clinical impact of HAA in AMI patients [5,6,23]. In the Translational Research Investigating Underlying disparities in acute Myocardial Infarction Patients’ Health Status (TRIUMPH) registry, Salisbury et al. reported an association of HAA with higher mortality and poorer health status in patients with AMI. In particular, patients with moderate to severe HAA had higher mortality rates and poorer health status at 1 year after adjustment for multivariable confounders, but no relationship was noted between mild HAA and mortality in these patients [5]. They also reported an independent correlation between HAA and acute renal failure/CKD. In the same registry, Salisbury et al. also found that HAA patients with persistent anemia 1 month after discharge had poorer health status and higher mortality than those in whom anemia had resolved [23]. These findings emphasize the clinical importance of HAA and indicated that further attention should be paid to HAA prevention, identification, and management. Although these studies also demonstrated a relationship between HAA and AKI/CKD and adjusted for CKD in the statistical model, they focused on the clinical outcomes of HAA in terms of severity and reversibility. The strong association for the development of HAA and the independent prognostic effect of AKI and CKD in AMI patients underscores the importance of understanding the clinical impact of HAA, especially when associated with AKI and CKD [27–30]. The finding that HAA patients with AKI and/or CKD had poorer prognostic outcomes than those without renal disease is an important and novel finding of the present study, and suggests that AKI and CKD are related to increased mortality risk in AMI patients who develop anemia during hospitalization. Several mechanisms may explain this. Anemia increases peripheral and myocardial tissue hypoxia, enhances the levels of pro-inflammatory cytokines, leads to LV dilatation and eccentric remodeling and activates the sympathetic nervous system and renin-angiotensin-aldosterone axis [31,32]. These changes might be even more prominent in the setting of decreased renal function such as that observed in case of AKI and CKD, and the observation of a graded increase in the risk of death when AKI and CKD exist simultaneously supports this assumption.

The present study has several limitations. We could only obtain limited information on several aspects due to the methodological limitations of retrospective analysis. The limited information available on the possible causes of AKI, CKD, and HAA, without details on the potential mechanisms, makes it impossible to definitively determine a causal relationship between AKI, CKD, and HAA, or to determine whether this is simply a marker for increased risk of death in AMI patients with HAA. HAA may have preceded AKI, and a temporal relationship between AKI and HAA is difficult to prove. In addition, proper interpretation of the results is limited by the absence of a pre-specified out-patient clinic visiting schedule, leading to lack of information on the persistence of anemia at a particular point after discharge. Moreover, unrecognized, minor bleeding events might not have been detected despite careful review of the medical records. However, the rate of bleeding complications was similar to previous reports [5,6]. More frequent serum Hgb measurements in patients with more severe symptoms represent another possible limitation, and this may increase the probability of detecting HAA. Finally, although we adjusted for multiple confounding factors, we cannot exclude the possibility of residual confounding factors due to the presence of an unmeasured confounder or measurement errors in the included factors.

In conclusion, we found that AKI and CKD are strongly associated with the development of HAA in AMI patients. The development of HAA is associated with higher long-term mortality when accompanied by AKI and/or CKD, even after adjustment for multivariable confounders. Our findings contribute to the understanding of the prognostic impact of HAA in AMI patients and can improve risk assessment in these patients.
